# Supplementary material for: Thiol-Ene Photopolymerization and 3D Printing of Non-Modified Castor Oil Containing Bio-Based Cellulosic Fillers
Source: Polymers (Basel). 2025 Feb 23;17(5):587. doi: 10.3390/polym17050587 (PMC11902828; doi:10.3390/polym17050587)
Supplement: Supplementary file 1 [file polymers-17-00587-s001.zip › polymers-3494578-supplementary.pdf]

Thiol-Ene Photopolymerization and 3D Printing of Non-Modified Castor Oil Containing Bio-Based Cellulosic Fillers

Support Information

Rafael Turra Alarcon [1], Matteo Bergoglio [2], Éder Tadeu Gomes Cavalheiro [1] and Marco Sangermano [2,*]

[1] Instituto de Química de São Carlos, Universidade de São Paulo-USP, São Carlos 13566-590, SP, Brazil
[2] Dipartimento Scienza Applicata e Tecnologia, Politecnico di Torino, Corso Duca degli Abruzzi 24, 10129 Torino, Italy
* Correspondence: marco.sangermano@polito.it

Contents

Figure S1. ^{1}H NMR of castor oil - page 2

Figure S2. Fillers used in this study – comparison of volume: (a) hemp, (b) walnut shell and (c) tagua - page 2

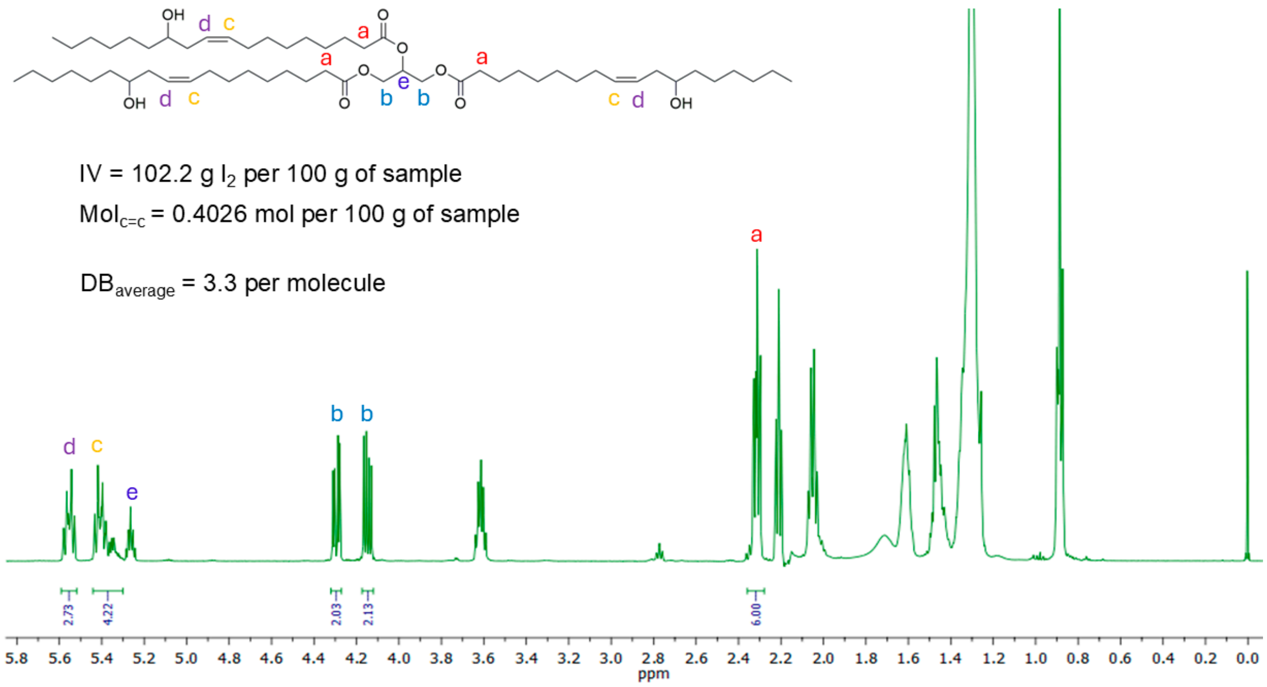

Figure S1. 1H NMR of castor oil.

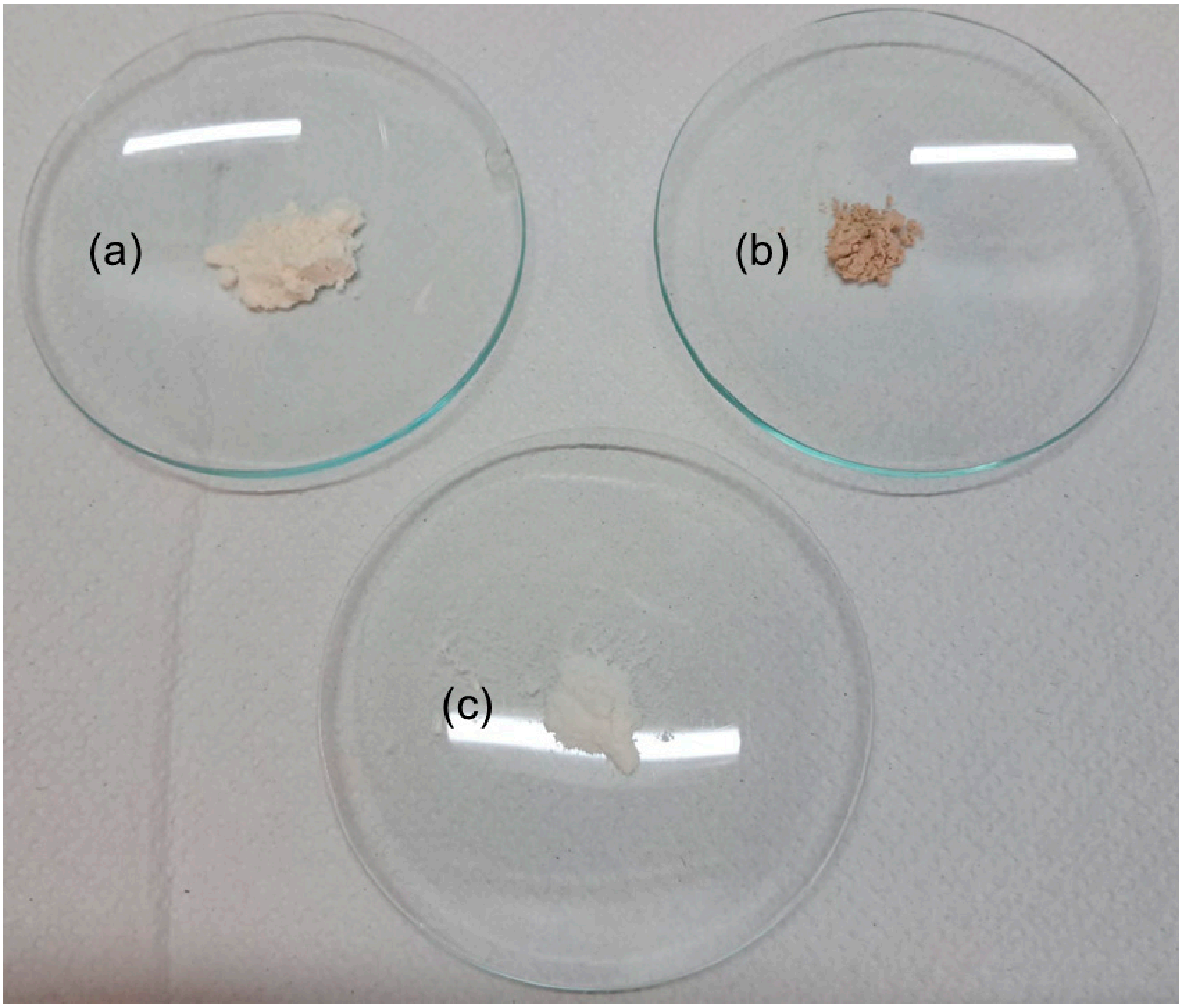

Figure S2. Fillers used in this study – comparison of volume: (a) hemp, (b) walnut shell and (c) tagua.
